# Controlling Topology of a Telomeric G‐quadruplex DNA With a Chemical Cross‐link

**DOI:** 10.1002/chem.202501467

**Published:** 2025-06-26

**Authors:** Bruce Chilton, Patrick J. B. Edwards, Geoffrey B. Jameson, Tracy K. Hale, Vyacheslav V. Filichev

**Affiliations:** ^1^ School of Food Technology and Natural Sciences Massey University Private Bag 11–222 Palmerston North 4442 New Zealand

**Keywords:** click‐chemistry, cross‐link, G‐quadruplex

## Abstract

DNA G‐quadruplexes (G4s) are noncanonical structures formed in guanine‐rich sequences. Within the human genome, they are nonrandomly distributed and influence DNA replication, gene expression, and genome maintenance. Numerous proteins involved in these processes have been identified as G4‐binding proteins. However, the interaction of proteins with G4s in the context of double‐stranded DNA in vitro has been difficult to study due to the transient nature of G4s in the presence of complementary DNA. To overcome this challenge, introducing internal covalent cross‐links between distant nucleotides within the DNA sequence may promote pre‐folding of G4 structures, thereby shifting the thermodynamic equilibrium toward G4‐formation. We used a Cu(I)‐catalyzed azide–alkyne cycloaddition to create a cross‐link between 2′‐*O*‐propargylguanosine and *N* ^6^‐azidoethyl‐2′‐deoxyadenosine in the DNA telomeric sequence (TAG_3_T)_2_. A cross‐link between G3 and A8 reinforced the parallel G4 topology that was stable in the presence of complementary DNA. Moreover, even in the presence of its complementary strand, this cross‐linked G4 recruited the parent native DNA (TAG_3_T)_2_ to form a hybrid G4. These results suggest that cross‐linking provides a useful tool for stabilizing noncanonical DNA structures in the presence of complementary strands, enabling their study within the context of genomic DNA.

## Graphical abstract

**Thermodynamically stable G‐quadruplex (G4) DNA**: Chemical cross‐linking between distant nucleotides led to formation of G4‐DNA that is stable in the presence of complementary DNA. This methodology can be used to reliably form G4s within DNA fibers for subsequent structural studies of G4‐protein interactions.

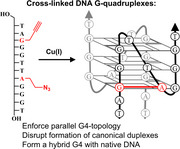

## 1 Introduction

**Figure 1:**
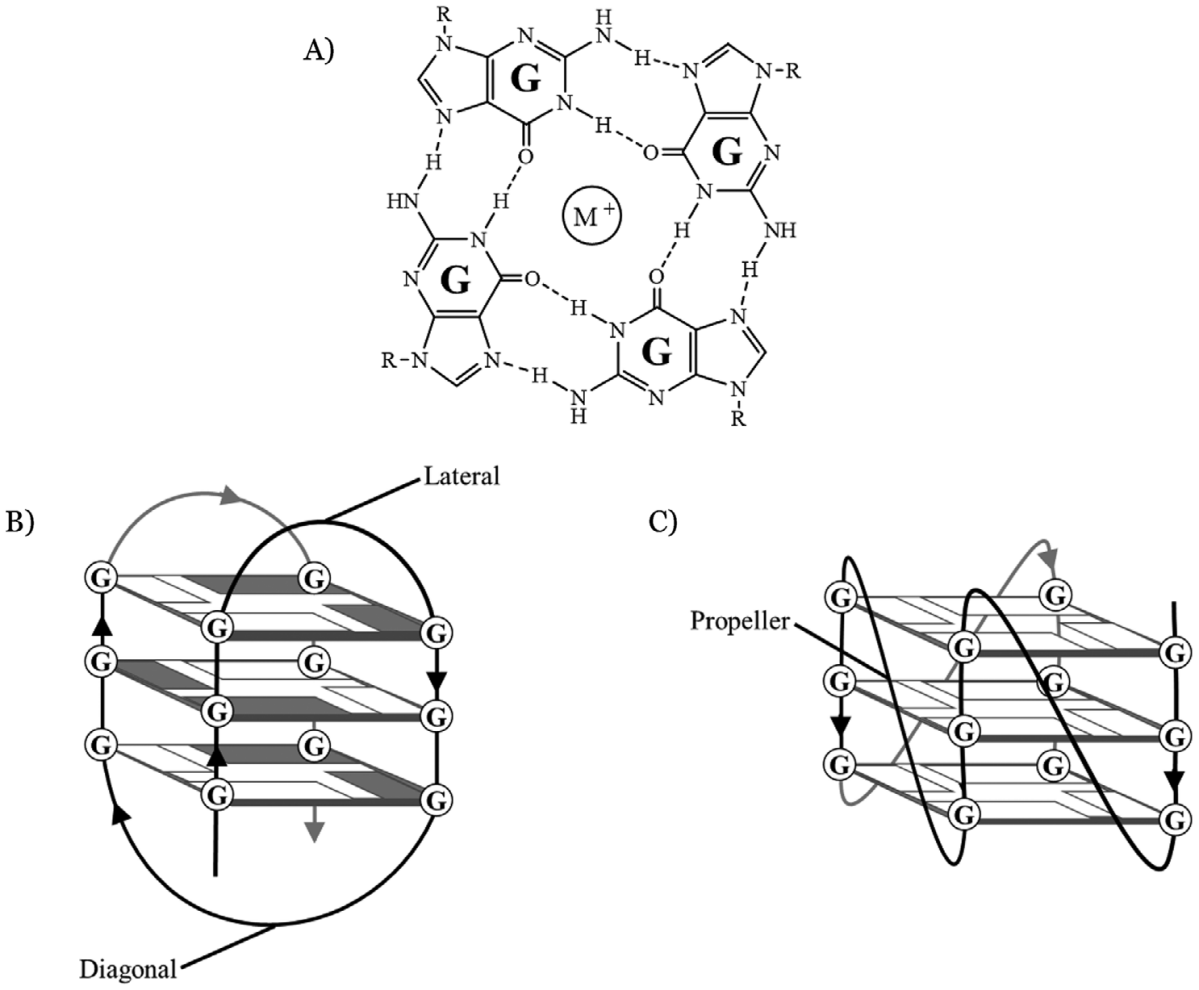
A) Hydrogen bonding arrangement of guanosine nucleobases to form a G‐tetrad arranged around a central cation, typically Na^+^ or K^+^. G‐tetrads stack to form either B) antiparallel G4 topologies, containing lateral and diagonal loops, or C) parallel G4 topologies, containing propeller loops. R = 2'‐deoxyribose in a G‐rich DNA sequence.

Within the genome, G‐quadruplexes (G4) have been shown to form in G‐rich regions of genomic DNA, generally with a sequence of (G_n_X)_4_, where X is any nucleotide and n is two or more. These noncanonical secondary structures occur when the G‐rich strand forms stacked G‐tetrads (Figure 1A), each consisting of four guanosine nucleotides arranged around a central monovalent cation such as Na^+^ or K^+^.^[^ ^1^, ^2^ ^]^ Enriched in regulatory regions and telomeres, the G4s formed in these G‐rich strands are recognized by proteins that play important roles in DNA replication and damage, transcription, genome organization and stability. This includes Heterochromatin Protein 1α (HP1α, responsible for forming and maintaining chromatin in cells),^[^ ^3^ ^]^ DNA methyltransferase 3A (DNMT3A, an enzyme responsible for DNA methylation)^[^ ^4^ ^]^ and Pif1 (a helicase that unwinds G4 structures in cells).^[^ ^5^ ^]^ However, for many their recognition of G4s and its functional importance is yet to be determined.

In vitro studies show that G‐rich oligonucleotides adopt G4s of various topologies depending on pH, salt concentration, and molecular crowding. In G4s of parallel topology, 2′‐deoxyguanosine glycosidic bonds adopt the *anti*‐conformation with all G‐tracts oriented in the same direction and connected by propeller loops while antiparallel structures have a mixture of 2′‐deoxyguanosines with *syn‐* and *anti‐*glycosidic bonds, with at least one G‐tract oriented in an opposite direction leading to lateral and diagonal loops (Figure 1B and C). In vitro binding assays have indicated that G4 binding proteins may have a preference for one topology over another, such as HP1α which prefers G4s of a parallel topology.^[^ ^3^ ^]^

However, studying the formation and topology of G4s within the context of DNA duplexes has been difficult given the preference of these G4 structures to form canonical DNA duplexes when complementary DNA is introduced.^[^ ^6^, ^7^ ^]^ This has hindered in vitro biophysical and structural characterization of proteins binding G4s within the DNA fiber.

While several approaches have been shown to stabilize G4s, they all present difficulties when generating a stable G4 located within duplex DNA. For example, although modified nucleotides such as 2′‐fluoroarabinonucleic acid, were able to kinetically trap G4s, the thermodynamic product was still duplex DNA.^[^ ^8^, ^9^ ^]^ Small‐molecule ligands and molecular‐crowding approaches could also shift the equilibrium toward G4s, but potentially alter the topology of the G4 under study and risks interfering with G4‐protein interactions.

**Figure 2:**
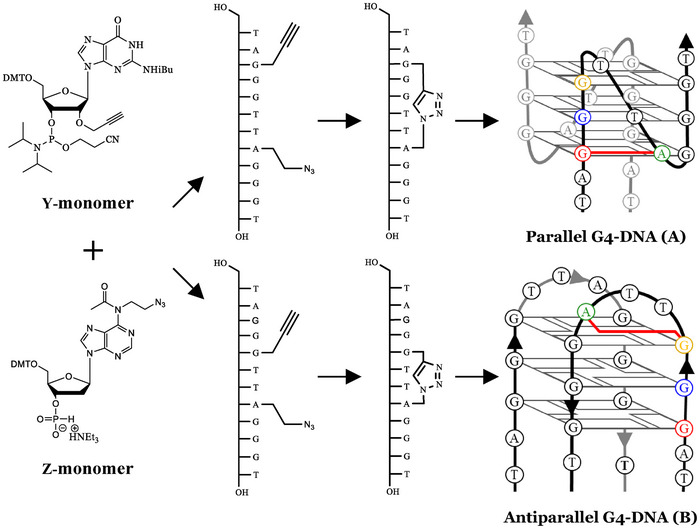
Modified nucleosides used to introduce 1,4‐disubstituted triazole cross‐links into G4 DNA. Top: commercially available 2′‐*O*‐propargylguanosine phosphoramidite (**Y**‐monomer); bottom: *N* ^6^‐modified adenosine H‐phosphonate containing azide (**Z**‐monomer). These modifications were incorporated during DNA synthesis and then cross‐linked post‐synthetically to form the thermodynamically trapped G4s shown. A G3A8 cross‐link in the 12‐mer telomeric sequence is proposed to reinforce the parallel G4 topology shown in (A) whereas a G5A8 cross‐link should form a G4 of antiparallel topology shown in (B).

Recently, we presented a method of stabilizing G4s in the presence of complementary DNA by inverting the polarity through 5′‐5′ or 3′‐3′ linkages within the G4 sequence.^[^ ^10^ ^]^ Here, we propose an alternative approach, introducing a cross‐link between two distant nucleotides, which cannot be broken, even when the structure unfolds. We hypothesized that cross‐links would promote formation of a specific G4 topology by pre‐forming parts of the G4 structure, as shown in Figure 2, resulting in increased thermal stability and resistance to unfolding in the presence of a complementary DNA sequence.

## 2 Methodology of Investigation

Using the mammalian telomeric repeat (TTAGGG)_n_, a 12‐mer G4‐forming sequence, tel (TAG_3_TTAG_3_T), was chosen to test the cross‐linking strategy. Previously NMR studies established that this DNA forms a parallel bimolecular G4 in K^+^‐containing buffer.^[^ ^11^, ^12^, ^13^ ^]^ The length of the sequence and the symmetrical nature of the resulting G4 assembly simplify the synthesis and analysis of G4s formed by ^1^H NMR.

Copper(I)‐catalyzed azide‐alkyne cycloaddition (CuAAC) was selected as the method of cross‐link formation for several reasons. A single cross‐link can be introduced using two modified nucleotides: one carrying an azide and the other a terminal alkyne, which together form a 1,4‐disubstituted triazole after CuAAC (Figure 2). The azide and alkyne functional groups are stable under the conditions of DNA synthesis, allowing modified nucleotides to be incorporated during DNA synthesis and cross‐linked post‐synthetically.^[^ ^14^, ^15^, ^16^, ^17^ ^]^

**Figure 3:**
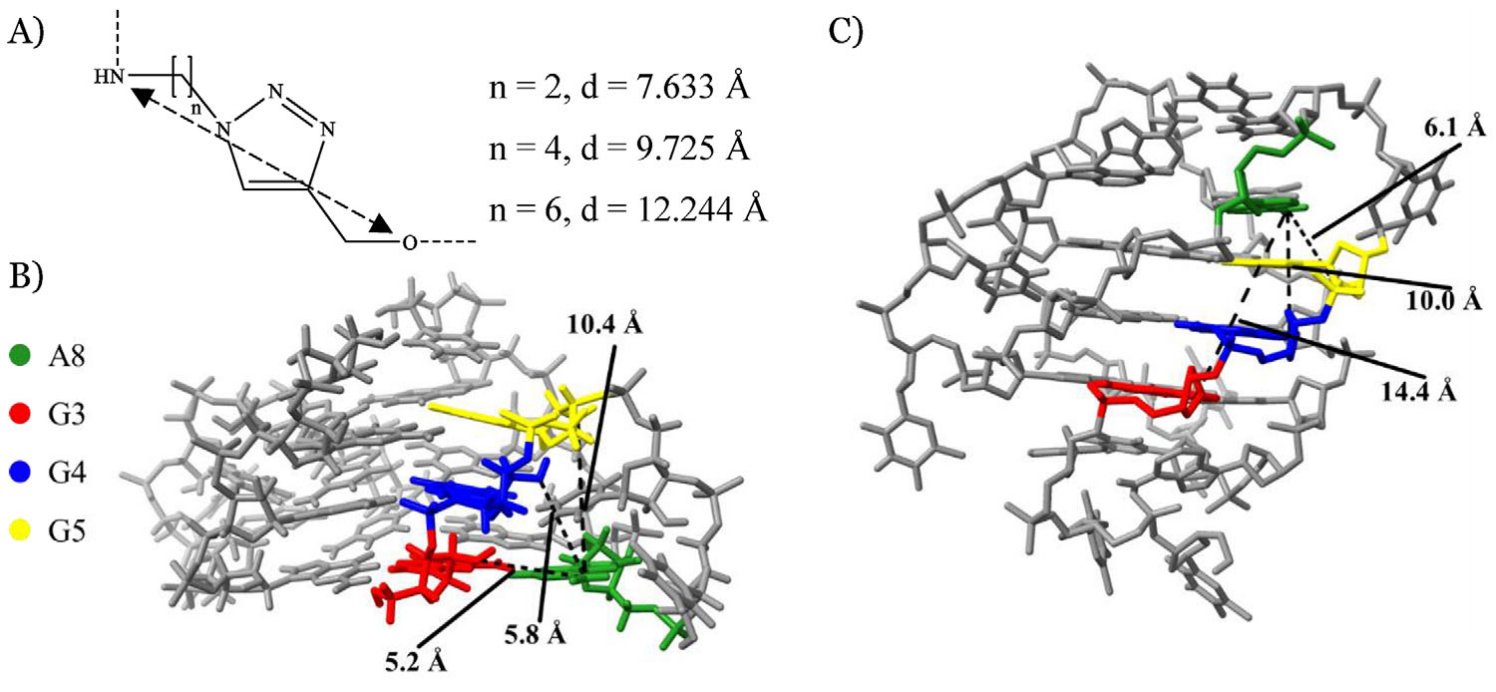
Choice of cross‐links for parallel and antiparallel G4‐DNA. A) Distance dependence between NH and O on the number of carbons in the proposed cross‐links formed by CuAAC. B) Parallel telomeric G4 structure, 2M18, indicating distances between potential modification sites. C) Antiparallel telomeric G4 structure, 2MBJ, indicating distances between potential modification sites.

Recently, we reported the use of CuAAC between distant nucleotides in the sequence to enforce the *U*‐shape observed in crystal structures for binding of single‐stranded linear DNA fragments with APOBEC3A enzyme, leading to better substrates and more potent inhibitors of APOBEC3A in comparison with linear DNA sequences.^[^ ^18^ ^]^ For this purpose, modified adenosine and uracil nucleosides had been obtained for cross‐linking. Given the abundance of guanosine nucleotides in G4‐forming sequences, the commercially available 2′‐*O*‐propargylguanosine phosphoramidite, shown in Figure 2, is a viable alternative to the modified phosphoramidite used previously for introducing the alkyne functionality (**Y** monomer in Table 1 and Figure 2). Introduction of an alkyne at the 2′‐position avoids interference with G‐tetrad formation. Previously, a 2‐carbon *N* ^6^‐azido adenosine (**Z** monomer) was used to introduce the azide functionality.^[^ ^18^ ^]^ Different linker lengths in the final triazole cross‐link were considered (Figure 3A), with the final nucleosides chosen based on the modification sites selected using NMR solution structures of parallel and antiparallel telomeric G4s (PDB: 2M18,^[^ ^19^ ^]^ Figure 3B, and 2MBJ,^[^ ^20^ ^]^ Figure 3C, respectively). When possible, an RNA structure (2M18) was used to consider the stereochemical influence due to the ribose nature of 2′‐*O*‐propargylguanosine.

**Table 1:** Unmodified and modified sequences based on the human telomeric repeat used in this study.

Name	DNA Sequence 5′ ‐ 3′		Retention Time (min)	*ESI‐MS [Da] found/calculated*
tel	TAG_3_T_2_AG_3_T	Linear	–	–
tel‐G3A8	TA**Y**G_2_T_2_ **Z**G_3_T	Linear	15.38	3878.649/3877.702
tel‐G3A8‐X		Cross‐linked	14.69	3878.667/3877.702
tel‐G5A8	TAG_2_ **Y**T_2_ **Z**G_3_T	Linear	15.62	3900.631(Na^+^)/3900.691(Na^+^)
tel‐G5A8‐X		Cross‐linked	14.46	3878.665/3877.702
c‐tel	AC_3_TA_2_C_3_TA	Linear	–	–

^[a]^ **Y** = 2′‐*O*‐propargylguanosine, **Z** = *N* ^6^‐azidoethyl‐2′‐deoxyadenosine. Sequence names containing ‐X indicate that Cu(I)‐catalyzed azide–alkyne cycloaddition has been performed, and a cross‐link has been formed. RP‐HPLC conditions: 0.1 M triethylammonium acetate buffer, pH 7 and acetonitrile (acetonitrile gradient overtime: 0 to 25% (0 – 20 min), 25 to 80% (20 – 22 min), 80% (22 – 24 min)).

Potential cross‐link sites to encourage either parallel or antiparallel G4 formation were identified by analyzing the distance between the 2′‐OH (or 2′‐H) of guanosines and *N* ^6^ positions of adenosines. These distances varied from 5.2 Å to 14.4 Å between G3 and A8 in parallel and antiparallel G4s, respectively. Hence, we predicted that a G3‐A8 or G4‐A8 cross‐link would form a parallel G4 (Figure 3B) whereas G4‐A8 and G5‐A8 cross‐links would form an antiparallel G4 (Figure 3C). Producing oligonucleotides to exclusively adopt only one topology required linkers that sufficiently restricted the G4 structure. Therefore, based on the distances shown in Figure 3, G3‐A8 (Figure 2A) and G5‐A8 (Figure 2B) were selected for parallel and antiparallel topologies, respectively. A two‐carbon linker was estimated to be approximately 7.6 Å based on Spartan calculations using 6–31 G* basis set (Figure 3A), which should only allow for the specific topology to form. Longer linkers such as four‐carbon (9.7 Å) and six‐carbon (12.2 Å) were rejected as they allowed too much flexibility that may result in formation of other topologies. Therefore, to assess the ability of the triazole linkers with the two‐carbon azide modification to control G4 topology we synthesized two DNA sequences and then cross‐linked them: G3‐A8 (TA(2′‐*O*‐propargyl‐G)G_2_TT(N_3_‐dA)G_3_T) and G5‐A8 (TAG_2_(2′‐*O‐*propargyl‐G)TT(N_3_‐dA)G_3_T) (see Table 1).

To introduce the azide functionality required for CuAAC, an unmodified adenosine could be replaced with a *N* ^6^‐position azide‐modified adenosine phosphoramidite (**Z** in Table 1) whose synthesis was reported previously,^[^ ^18^ ^]^ shown in Figure 2. Although phosphoramidites have a propensity to react with an azide in a Staudinger reaction^[^ ^21^ ^]^ this can be circumvented by using a nucleoside phosphoramidite with azide in DNA synthesis immediately after its formation with minimal work‐up and avoiding concentrating the phosphoramidite solution. An alternative approach is to convert this nucleoside into an H‐phosphonate, which can be purified, characterized and stored for extended time at ‐20 °C.^[^ ^22^, ^23^, ^24^ ^]^ Therefore, we prepared the H‐phosphonate of *N^6^*‐acetyl‐*N* *6*‐(2‐azidoethane)‐2′‐deoxyadenosine (see Supporting Information), which was incorporated along with 2′‐*O*‐propargylguanosine into the tel sequence at the selected positions during DNA synthesis (Table 1).

After folding, these sequences were evaluated based on topology, stability, and their impact on canonical duplex formation in the presence of complementary DNA. These can be assessed using ^1^H NMR and CD spectroscopies. In ^1^H NMR spectra we can analyze peaks corresponding to imino protons involved in hydrogen bonding to obtain accurate topological information by comparing the observed peaks of the modified sequences to the tel control. Canonical duplexes give imino proton peaks between 12 and 14 ppm, whereas G4 structures give imino protons between 10 and 12 ppm. Additionally, parallel G4 structures give a positive CD peak at 265 nm and negative at 240 nm. Antiparallel G4s have positive peaks at 290 and 240 nm and a negative peak at 265 nm.^[^ ^25^, ^26^, ^27^, ^28^ ^]^ This can be combined with ^1^H NMR data to confirm topologies of modified sequences. Thermal stability can be determined by heating samples stepwise, while recording CD and NMR spectra at specific temperatures. *T*_1/2_ is defined as the temperature at which half of the structure is unfolded, determined by comparing the intensity of the CD signal across the range of experimental temperatures to the maximum and minimum CD signal intensities, as described in the experimental section.

## 3 Results and Discussion

### 3.1 Confirming Formation of Cross‐links

Modified tel sequences listed in Table 1 were synthesized, purified by reverse‐phase HPLC (RP‐HPLC, Figure SI‐1A and B), and then cross‐linked using the conditions described in the Supporting Information and Table SI‐1.^[^ ^29^ ^]^ Successful cross‐link formation was determined using RP‐HPLC where a decrease in retention time of approximately 1 minute was consistently observed for the cross‐linked DNA in comparison with the linear DNA (Figures SI‐1C and D, respectively). This agrees with earlier observations for cross‐linked products.^[^ ^18^, ^30^, ^31^ ^]^ The cross‐linked products were purified with RP‐HPLC (Figures SI‐1E and F). Their composition was confirmed by ESI‐MS (Figure SI‐2) and by ^1^H NMR, where the disappearance of the peak corresponding to the alkyne proton (approx. 2.1 ppm) and shifts in peaks corresponding to 2′‐OH (approx. 1.6 ppm) and the CH_2_ of the alkyne modification (approx. 3.4 ppm) also confirmed formation of a triazole linkage (Figure SI–3). To distinguish cross‐linked from linear sequences in the text, X is used at the end of the DNA name for the cross‐linked product (Table 1).

### 3.2 Formation and Topology of G4s Containing Cross‐Links

^1^H NMR and circular dichroism spectroscopies were used to determine the topological features of complexes formed by the modified oligonucleotides and compared to the unmodified tel sequence.

**Figure 4:**
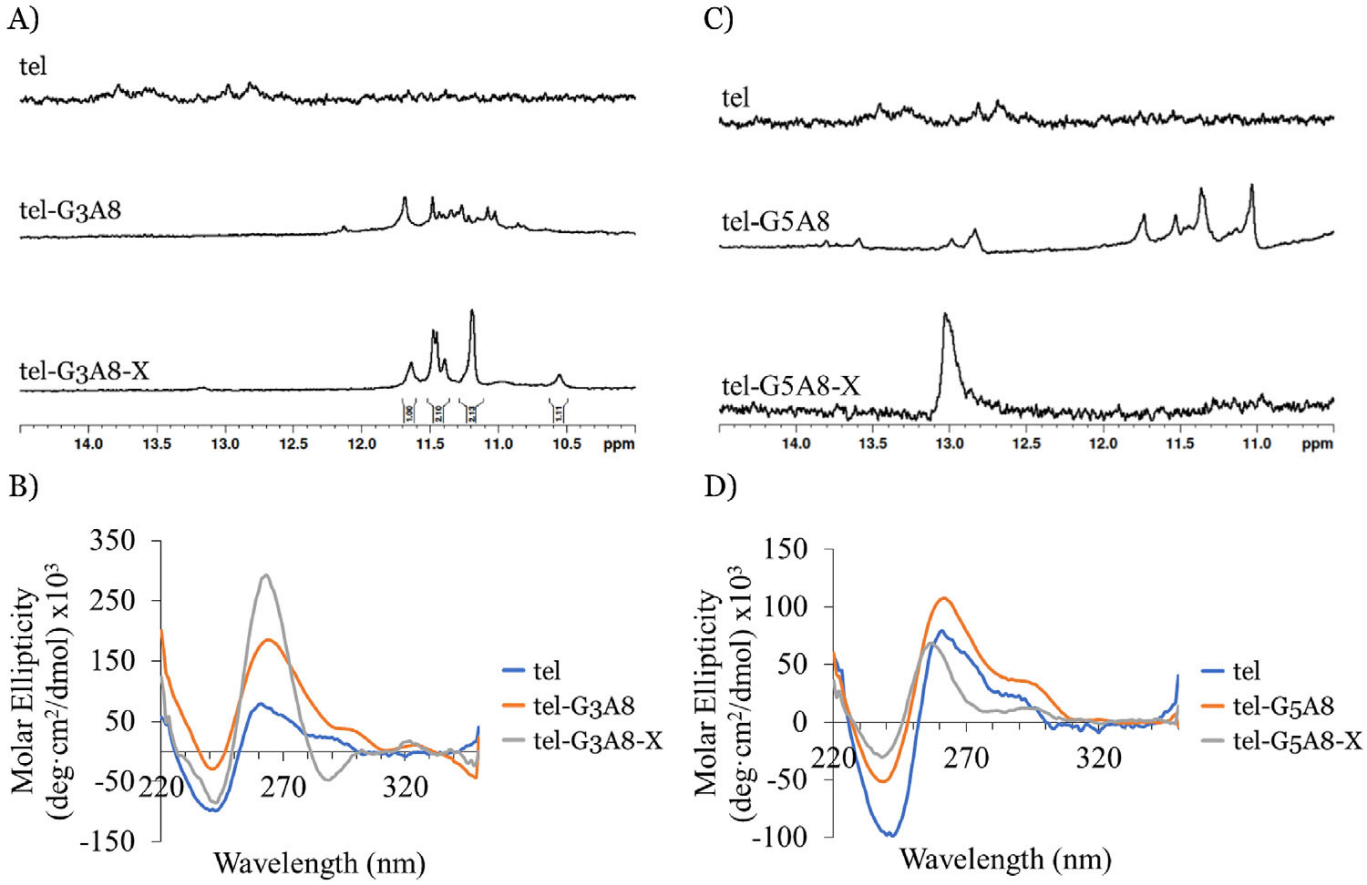
Analysis of topological changes induced by cross‐links in G4‐forming sequences based on the human telomeric repeat in K^+^ buffer. NMR (A) and CD (B) profiles of tel‐G3‐A8. NMR (C) and CD (D) profiles of tel‐G5‐A8. Conditions: 20 µM (CD) or 200 µM (NMR) strand concentration, 20 mM sodium phosphate, 10 mM KCl, 10% D_2_O, 1% trimethylsilylpropanoic acid (TSP), pH 7.0, 20 °C.

The unmodified DNA tel sequence remains unfolded in Na^+^‐ and K^+^‐containing buffers as evidenced by the absence of significant imino proton peaks in ^1^H NMR spectra at 20 °C (Figure 4A and Figure SI–4B in the Supporting Information). Some low‐intensity peaks appeared between 13 and 14 ppm, indicative of partial formation of canonical Watson‐Crick base pairs rather than G4 formation. In contrast, tel‐G3A8 and its cross‐linked product, tel‐G3A8‐X, formed G4‐DNA in both buffers with more clearly distinguishable imino proton peaks appearing between 10.5 and 12 ppm in K^+^ buffer. Integration of imino proton peaks in tel‐G3A8‐X spectrum indicated that six imino protons of guanosine were involved in hydrogen bonding (twelve if we assume diagonally opposed nucleotides are chemically equivalent), suggesting that a single topology is formed rather than the mixture of topologies present in the non‐cross‐linked sample. These observations were also supported by CD spectra as the peak with positive ellipticity at 260 nm for unstructured tel sequence shifted to 265 nm for tel‐G3A8 and tel‐G3A8‐X in K^+^ buffer (Figure 4B). This was also accompanied by increased intensity of this peak for modified sequences which is characteristic for formation of G4‐DNAs of parallel topology. Similar effects but with lower peak intensity enhancement were seen in Na^+^ buffer (Figure SI–4A in Supporting Information). The tel control is reported to form a more thermally stable G4 at higher concentration of K^+^ ions, but the presence of several broad peaks in the imino region indicates the formation of multiple species in solution.^[^ ^13^ ^]^ In this case, the introduction of the cross‐link had the anticipated effect of encouraging formation of the parallel G4. Compared to the mostly unfolded tel and tel‐G3A8 sequences, the cross‐linked sequence was significantly more structured in Na^+^ buffer, suggesting that cross‐linking was sufficient to encourage G4 formation independent of the cations used.

However, different results were obtained for G5A8 modifications in the telomeric sequence for both Na^+^ and K^+^ buffers. While tel‐G5A8 sequence showed formation of low‐intensity imino proton peaks in the 11–12 ppm region that corresponds to G4 formation, the cross‐linked tel‐G5A8‐X sample had a single peak at 13.0 ppm, which is a sign of formation of A‐T or G‐T base pairs rather than G4 hydrogen bonds (Figure 4C).^[^ ^32^ ^]^ This suggests that the cross‐link between G5 and A8 removed the ability of the sequence to form G4s and caused the sequence to instead adopt a hairpin‐like structure. It is notable that peaks at 12.8–13.0 ppm, although of low intensity, are present in the tel and tel‐G5A8 samples suggesting that the cross‐link traps the sequence in one of its non‐G4‐conformations. CD spectra of tel‐G5A8 sequences also indicated a limited ability to form G4s, indicated by peaks with positive ellipticity for tel‐G5A8 at 260 nm and tel‐G5A8‐X at 257 nm, which are typical of single‐stranded DNA with high guanine content.^[^ ^27^ ^]^

### 3.3 Thermal Stability of G4s Containing Triazole Cross‐Links

Next, we evaluated G4 stability by performing melting studies and monitoring changes in CD spectra with increased temperature. Assessing the impact of cross‐linking on G4 stability in Na^+^‐containing buffer is challenging due to the relatively low stability of the unmodified species. Only the cross‐linked tel‐G3A8‐X formed a G4 in Na^+^ buffer, with a *T* **_½_** value of 54 °C (Table 2, Figure SI–5). However, as no structure was formed for unmodified sequences, the cross‐linked structure appears to be more stable than the native structure.

**Table 2:** Melting temperatures of telomeric oligonucleotides with and without cross‐links^[^ ^a^ ^]^.

Name	*T*_½_ (K^+^, °C, ± 2 °C)
tel	Unfolded
tel‐G3A8	58
tel‐G3A8‐X	42
tel‐G3A8‐X (Na^+^)^(a)^	54
tel‐G5A8	50
tel‐G5A8‐X	51

^[a]^ This is the only sequence that forms a stable G4 in Na^+^‐containing buffer. Conditions: 20 µM strand concentration, 20 mM sodium phosphate, 10 mM KCl, pH 7.0.

In contrast to the unfolded tel sequence in K^+^ buffer, the oligonucleotides with G3 and A8 modifications gave a *T* **_½_** of 58 °C for tel‐G3A8 and, surprisingly, 42 °C for tel‐G3A8‐X (Table 2, SI–6B and C). Although the samples differed slightly in topology, the non‐cross‐linked tel‐G3A8 structure appears to be more thermally stable than the structure supported by the cross‐link in K^+^‐containing buffer. Interestingly, the cross‐linked tel‐G3A8‐X structure was less thermally stable in K^+^ buffer than in Na^+^ buffer despite a G4 of more distinct topology being formed in K^+^ buffer, according to ^1^H NMR spectroscopy at 20 °C in K^+^ buffer. This unexpected conclusion is also supported by the similarity in *T* **_½_** of tel‐G5A8 and tel‐G3A8 structures. We note that native tel was reported to have a *T* **_½_** value of 41 °C at higher strand and K^+^ concentrations (respectively, 75 µM and 10 mM).^[^ ^13^ ^]^ Further enhancement in thermal stability (Δ*T* **_½_** = 6 °C) was observed when guanosine G9 was in its ribose form, which reflects a similar stabilizing effect for tel‐G3A8 and tel‐G5A8 in comparison to tel sequence.^[^ ^13^ ^]^ Both tel‐G5A8 and tel‐G5A8‐X were unfolded in Na^+^ buffer, and only tel‐G5A8 is folded into a G4 in K^+^ buffer, where its *T* **_½_** is 50 °C, slightly lower than both tel and tel‐G3A8 structures (Figure SI–6D). Although tel‐G5A8‐X does not appear to form a G4, the unique hairpin secondary structure that is formed is remarkably stable given the limited base‐pairing, with a *T* **_½_** of approx. 51 °C (Figure SI–6E).

### 3.4 Competition of Cross‐Linked G4s with Canonical Duplex Formation

**Figure 5:**
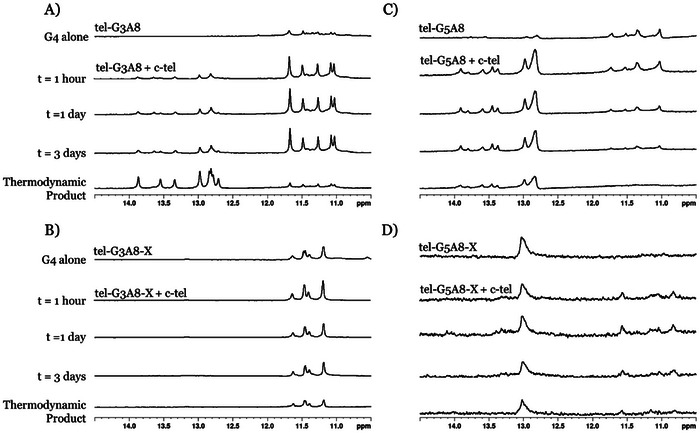
^1^H NMR of telomeric G4 sequences containing CuAAC cross‐links challenged with complementary DNA. A) tel‐G3A8 + c‐tel. B) tel‐G3A8‐X + c‐tel. C) tel‐G5A8 + c‐tel. D) tel‐G5A8‐X + c‐tel. Conditions: 200 µM strand concentration, 20 mM sodium phosphate, 10 mM KCl, 10% D_2_O, 1% TSP, pH 7.0, 20 °C.

Duplex formation of modified telomeric sequences was studied by challenging each modified tel sequence with complementary DNA and observing the changes in the imino proton region using ^1^H NMR spectroscopy at 20 °C. The unmodified control forms a duplex immediately upon addition of the complementary strand and within one day it is completely folded into a duplex (Figure SI‐7). On the other hand, tel‐G3A8 only partially formed a duplex after three days (approx. 20% duplex formation by integrating both regions) and required heating to 90 °C and cooling down to room temperature before the duplex became the dominant structure (Figure 5A). There is precedent in previously reported experiments with 2′‐fluoroarabinonucleic acid that modified nucleotides can lead to kinetically trapped G4‐DNA.^[^ ^9^, ^13^ ^]^ Here, the very slow conversion at 20 °C of G4 tel‐G3A8 to the thermodynamically stable duplex indicates a significant kinetic barrier. In marked contrast, the G4 formed by the cross‐linked tel‐G3A8‐X sequence was still present after the sample was heated and cooled down to room temperature with no observation of ^1^H NMR peaks corresponding to the duplex formation (Figure 5B). Thus, in contrast to tel‐G3A8, for tel‐G3A8‐X the G4 structure is thermodynamically favored over the duplex structure at 20 °C when these oligonucleotides are challenged with the complementary sequence. Moreover, modified oligonucleotides by themselves do not bestow thermodynamic stability of G4 over duplex structures; rather, thermodynamic stability requires the triazole cross‐linked species, tel‐G3A8‐X. This result is not a contradiction of the reduced thermal stability of tel‐G3A8‐X compared to tel‐G3A8 as those measurements were conducted in the absence of complementary DNA.

Upon addition of complementary DNA, the tel‐G5A8 sequence behaved similarly to the tel control. We observed the immediate appearance of peaks in the ^1^H NMR spectrum corresponding to the duplex (Figure 5C). After three days approximately 75% conversion to duplex had occurred (determined by integrating both regions). Complete conversion to the thermodynamically favored duplex required heating and cooling of the tel‐G5A8 structure. This suggests that shifting the position of 2′‐*O*‐propargylguanosine from G3 to G5 somewhat disfavored formation of kinetically trapped G4. In contrast to tel‐G3A8‐X, the cross‐linked tel‐G5A8‐X sequence formed neither a duplex nor a G‐quadruplex structure in the presence of a complementary strand according to NMR spectroscopy (Figure 5D), suggesting that the cross‐link stabilized a DNA hairpin. As tel‐G3A8‐X retained a parallel G4 structure in the presence of complementary DNA, our hypothesis that specific secondary structures can be stabilized by incorporating a cross‐link between distant nucleotides is sustained. However, selection of appropriate modification sites is clearly crucial for stabilizing G4 structures.

**Figure 6:**
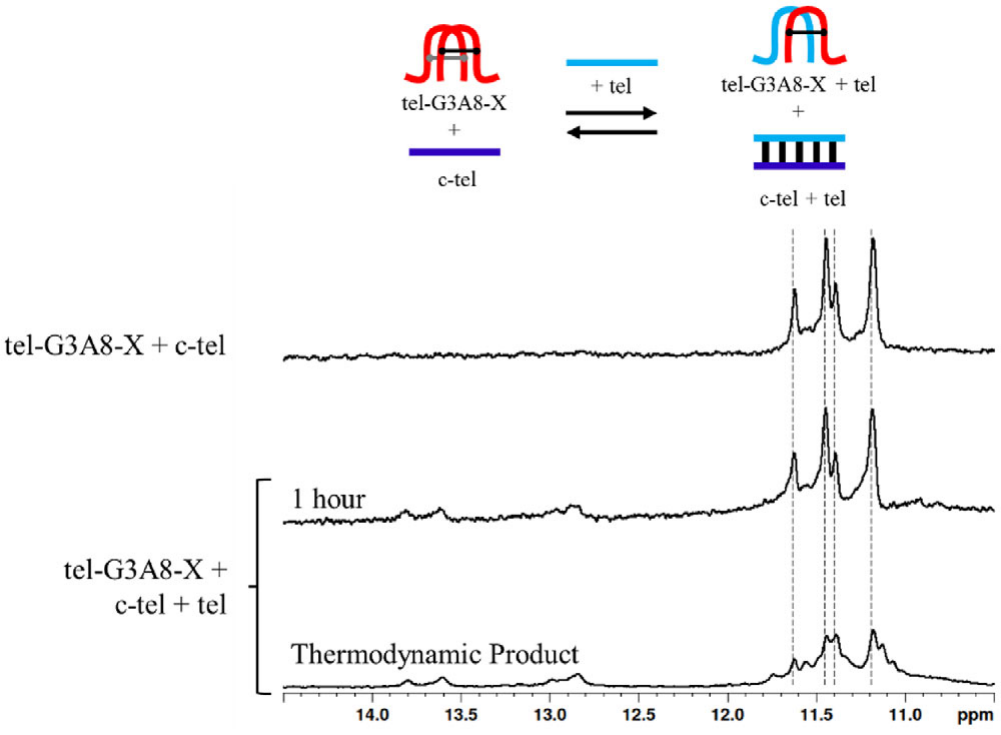
Addition of tel to tel‐G3A8‐X and c‐tel mixture, indicating formation of canonical duplex and tel‐G3A8‐X/tel G4 hybrids. Conditions: 200 µM tel‐G3A8‐X and c‐tel strand concentration, 150 µM tel strand concentration, 20 mM sodium phosphate, 10 mM KCl, 10% D_2_O, 1% TSP, pH 7.0, 20 °C. Dashed lines indicate peaks of tel‐G3A8‐X G4, which are still present following addition of tel.

To investigate the potential to form a hybrid G4 structure between cross‐linked tel‐G3A8‐X and native tel sequence (TAG_3_TTAG_3_T), 0.75 molar equivalent of tel was added to the solution containing a 1:1 mixture of tel‐G3A8‐X and c‐tel. The products of this experiment are shown in Figure 6. Initially, minimal changes were observed to the imino proton peaks around 11.5 ppm, corresponding to the G4 formed by tel‐G3A8‐X, but low‐intensity peaks between 13 and 14 ppm appeared, indicating some duplex formation between tel and c‐tel (Figure 6). After heating this sample to 90 °C, cooling slowly and storing at 4 °C overnight, additional peaks have appeared in the range of 11–11.8 ppm (Figure 6). This suggests that more than one G4 structure is present, or that the resulting G4 structure is no longer symmetrical. Given that tel does not form a stable G4 in these conditions and quickly forms a duplex with the complementary strand and that the tel‐G3A8‐X in presence of c‐tel shows no change under this heating and cooling regime, the changes in the imino proton region around 11 pm support the formation of an asymmetrical, hybrid G4 composed of tel‐G3A8‐X and tel sequences, along with the original tel‐G3A8‐X G4 structure (noting that tel was added at only 0.75 mole‐equivalent to c‐tel and tel‐G3A8‐X). The presence of imino proton peaks in the region of canonical base‐pairs suggests that tel forms a canonical duplex with the complementary c‐tel. To evaluate the propensity of tel to form duplex with c‐tel, we compared integration of peaks for canonical G‐C base‐pairing (12.6–13.3 ppm) and G‐tetrads (10.8–12.0 ppm) relative to the internal standard (TSP, 0.0 ppm). The number of protons engaged in noncanonical hydrogen bonding (G4) remains constant following addition of tel to tel‐G3A8‐X (0.5 H), but the number of protons engaged in canonical G‐C base‐pairing (0.08 H) is equivalent to only 10% of the tel/c‐tel duplex (0.8 H, Figure SI–7), despite adding 0.75 eq. of tel, both before and after the heating‐cooling regime. This suggests that an equilibrium exists between the canonical tel/c‐tel duplex, the original G4 of tel‐G3A8‐X and a hybrid‐G4 of tel‐G3A8‐X/tel. Remarkably and to our surprise, under conditions where tel does not form a G4 species, in the presence of tel‐G3A8‐X, the dodecameric tel prefers to form a hybrid G4 with tel‐G3A8‐X than a duplex with its complementary strand.

## 4 Conclusions and Perspectives

We have reported the incorporation of azide and alkyne modifications into a short G4‐forming telomeric sequence and the subsequent creation of novel cross‐linked G4 structures using copper(I)‐catalyzed azide–alkyne cycloaddition. Non‐cross‐linked sequences resulted in similar structures and stabilities to unmodified DNA and created kinetically stable G4 structures with similar properties to existing modifications, such as 2′‐fluoroarabinonucleic acids.^[^ ^9^ ^]^

The cross‐linked tel‐G3A8‐X sequence formed a G4 of parallel topology, even in Na^+^ buffer, but this had decreased thermal stability compared to the unmodified and non‐cross‐linked G4s. Despite this, the cross‐linked G4 was thermodynamically stable when challenged with the complementary strand. Furthermore, when the unmodified tel sequence was added to the cross‐linked G4 in the presence of tel's complementary sequence, tel and tel‐G3A8‐X formed a hybrid G4, which was the dominant product in a mixture with the canonical duplex. This suggests that cross‐linked G4 promoted and stabilized G4 formation of the native sequence providing an interesting example of induced‐fit recognition between chemically modified and native G4s. Conversely, the tel‐G5A8‐X sequence, intended to create an antiparallel G4, formed instead a DNA hairpin that was also resistant to addition of complementary DNA.

Overall, these results suggest that cross‐links between distant nucleotides in DNA can be an effective method of reinforcing an existing topology with several key considerations. Firstly, careful positioning of modifications is critical to controlling the resulting topology. When positioned correctly they can enforce G4 and disrupt duplex formation, but when positioned incorrectly they may also disrupt G4 formation in favor of another topology such as a DNA hairpin. Secondly, longer linkers between nucleotides could provide greater flexibility to the structure, allowing both for formation of alternative topologies and better optimization of H‐bonds to improve thermal stability. However, this also risks removing restrictions on the structure that then favor duplex formation. Further optimization of linkers as well as testing a wider range of G4‐ or other secondary structure‐forming sequences is of interest to the authors. This includes investigation of cross‐linked sequences for drug development targeting G4‐specific proteins, but further testing of modification sites to select specific G4 topologies is necessary.

## 5 Experimental Section

The oligonucleotide controls used in this study were obtained from Integrated DNA Technologies. These oligonucleotides were dissolved in milli‐Q water and G4 structures were formed by diluting the stock solutions in 20 mM NaH_2_PO_4_/ Na_2_HPO_4_ buffer. Potassium chloride (10 mM) was added when required. Samples were annealed by heating at 90 °C for 5 minutes, cooling slowly to 4 °C, and storing for 24 hours at 4 °C.

DNA synthesis was carried out using a Mermade‐4 DNA/RNA automated synthesizer. Controlled pore glass supports carrying the first nucleoside were obtained from DNAture Diagnostics and Research Ltd (New Zealand). Standard 5′‐*O*‐DMT‐3′‐*O*‐phosphoramidites of nucleosides were obtained from Innovassynth Technologies (I) Ltd (India) and the 2′‐*O*‐propargylguanosine phosphoramidite was obtained from Chemgenes Corporation (USA). The coupling time of modified phosphoramidites was increased to 10 minutes. The protocol used for H‐phosphonate coupling is described in the Supporting Information. After the synthesis, oligodeoxynucleotides were cleaved from the solid support with 28% aq. ammonia at 55 °C for 12 hours. Ammonia was evaporated using an Eppendorf Concentrator Plus and oligodeoxynucleotides were purified using Thermo Scientific UltiMate 3000 UHPLC with an Alltech 250 mm × 4.6 mm, 10 µm Hypersil Gold column (Reverse‐Phase) or a TSKgel SuperQ‐5PV 7.5 mm I.D. × 7.5 cm, 10 µm column (Ion‐Exchange). Purified oligodeoxynucleotides were desalted using NAP‐5 size exclusion columns. Sequences were verified using mass spectrometry recorded in 15% methanol/H_2_O using electrospray ionization MS with a Thermo Scientific Q Exactive Focus Hybrid Quadrupole‐Orbitrap Mass Spectrometer. Masses are reported in atomic mass units (a.m.u.).

Circular dichroism (CD) spectra were recorded using a Chirascan CD spectrophotometer (150 W Xe arc) from Applied Photophysics with a Quantum Northwest TC125 temperature controller. Oligodeoxynucleotides were diluted to 20 µM in the reported buffers and placed in the 1 mm quartz cuvette. Scans were taken over a range of 220–350 nm with a bandwidth of 1 nm and response of 0.25 s. A buffer‐only baseline was subtracted from each CD spectrum before they were smoothed by averaging 10 neighbor points using software provided by Applied Photophysics Ltd. Melting experiments were performed by recording CD spectra every 2.5 °C with equilibration for 2.5 minutes at each temperature from 15 to 90 °C. The signal at maxima and minima was assessed and values were converted to fraction folded (θ_T_) using the formula:

$$\theta_{T}=\left(\theta -\theta_{\min}\right)/\left(\theta_{\max}-\theta_{\min}\right)$$

where θ is the CD signal at that temperature while θ_max_ and θ_min_ are the signals for completely folded and completely unfolded species, respectively. *T*_½_ is the temperature at which half of the structure is unfolded (θ = 0.5). *T*_½_ is not reported for sequences which did not completely unfold within the specified temperature range.

^1^H NMR spectra were recorded on a Bruker 700 MHz spectrometer with three‐channel cryoprobe using trimethylsilylpropanoic acid (TSP) as an internal standard at 20 °C. Chemical shifts are reported in parts per million (ppm) downfield of TSP. Spin multiplicities are described as: s (singlet), br.s. (broad singlet), d (doublet), dd (doublet of doublets), t (triplet), q (quartet), m (multiplet). Coupling constants are reported in Hertz (Hz).

## Supplementary Information

Supplementary experimental details about the synthesis of modified oligodeoxynucleotides and RP‐HPLC profiles and HRMS (ESI) spectra of oligodeoxynucleotides, CD spectra, DNA melting experiments.

## Author Contributions

B.C., G.B.J., T.K.H., and V.V.F. designed the research, B.C. performed the synthesis and purification of oligodeoxynucleotides and the CD experiments; P.J.B.E. and B.C. performed NMR experiments. All authors analysed the data; B.C. wrote the first draft of the article, all authors have read and agreed to the published version of the manuscript.

## Conflict of Interests

Authors declared no conflict of interests.

## Supporting information



Supplementary Information

## Data Availability

The data that support the findings of this study are available in the supplementary material of this article.
