# Supplementary material for: Controlling Topology of a Telomeric G‐quadruplex DNA With a Chemical Cross‐link
Source: Chemistry. 2025 Jun 26;31(40):e202501467. doi: 10.1002/chem.202501467 (PMC12272027; doi:10.1002/chem.202501467)
Supplement: Supplementary file 1 — Supplementary Information [file CHEM-31-e202501467-s001.docx]

Supporting Information for

**Controlling Topology of a Telomeric G-quadruplex DNA with a Chemical Cross-link**

Bruce Chilton, Patrick J. B. Edwards, Geoffrey B. Jameson, Tracy K. Hale and Vyacheslav V. Filichev*

School of Food Technology and Natural Sciences, Massey University, Private Bag 11–222, Palmerston North 4442, New Zealand

**Synthesis of azide-containing 2’-deoxyadenosine H-phosphonate**

**5’-*O*-(4,4’-Dimethoxytrityl)-*N^6^*-acetyl-*N^6^*-(2-azidoethane)-2’-deoxyadenosine 3’-H-phosphonate TEA salt (7)**

5’-*O*-(4,4’-Dimethoxytrityl)-*N^6^*-acetyl-*N^6^*-(2-azidoethane)-2’-deoxyadenosine^12^ (0.29 g, 0.44 mmol, 1 eq) was dissolved in 5 mL of pyridine and diphenyl H-phosphonate (0.71 g, 3.1 mmol, 7 eq) was added. The mixture was stirred at room temperature for 15 mins, monitored by TLC (9:1 Chloroform/methanol). When TLC analysis indicated reaction was complete, 1 mL of water and 1 mL of Et_3_N were added and the mixture was left for 15 mins. Volatile solvent was evaporated under reduced pressure. 20 mL of DCM was added and it was extracted three times with 20 mL of sat. NaHCO_3_. Solution was dried over anhydrous sodium sulfate, filtered, and evaporated under reduced pressure. Product was purified by silica gel column chromatography (0 – 12% MeOH/DCM + 1% Et_3_N) and obtained as a white foam. Yield: 0.31 g (85%)

^1^H NMR (500 MHz, d_6_-DMSO) δ = 8.73 (1H, s, H-8), 8.71 (1H, s, H-2), 7.36 – 7.32 (2H, m, DMT aromatic CH), 7.24 – 7.18 (7H, m, DMT aromatic CH (4H + 2H +1H)), 6.81 (4H, t, J = 9.8 Hz, DMT aromatic CH), 6.50 (1H, t, 6.7 Hz, H-1’), 4.82 (1H, m, H-3’), 4.30 (2H, t, J = 6.27 Hz, H-5’), 4.21 (1H, m, H-4’), 3.73 (6H, s, DMT O-CH_3_), 3.05 – 2.98 (1H, m, 2_a_’) 2.58 – 2.54 (1H, m, 2_b_’), 1.97 (3H, s, N-C(=O)-CH_3_), 1.24 (9H, t, J = 7.22 Hz, HN^+^CH_2_CH_3_).

^13^C NMR (125 MHz, d_6_-DMSO) δ = 170.73 (O=C-CH_3_), 158.46, (C-6), 152.94 (C-4), 152.05 (C-8), 145.28 (DMT-q), 145.09 (C-2), 136.08 (DM-q), 135.95 (DMT-q), 130.12 (DMT CH), 130.09 (DMT CH), 128.11 (DMT-CH), 127.60 (C-5), 127.01 (DMT CH), 113.50 (DMT CH), 86.47 (C-4’), 85.85 (DMT-q), 84.30 (C-1’), 70.83 (C-3’), 64.73 (C-5’), 55.44 (O-CH_3_ (DMT)), 55.43 (O-CH_3_ (DMT)), 50.91 (C-6’’), 46.72 (C-1’’), 39.10 (C-2’), 28.47/28.35 (C-2’’/5’’), 26.07/26.01 (C-3’’/4’’), 24.23 (N-C(=O)-CH_3_).

^31^P NMR (125 MHz, CDCl_3_) δ = -0.03108 (H-Phosphonate).

ESI-MS (MeOH, negative): Calculated: 727.24 . Experimental: 685.09 [M – acetyl, 15%], 727.09 [M, 100%].

**H-Phosphonate Coupling**

The H-phosphonate was dissolved in a dry pyridine/ACN (30/70) to make a 0.1 M solution, which was attached to an unused phosphoramidite port on a DNA synthesiser. Pivaloyl chloride (0.5 M) was dissolved in dry ACN and attached to a different unused phosphoramidite port on a DNA synthesiser. The solutions were mixed on the column using a wobble base protocol to ensure adequate mixing of the H-phosphonate with the activator. Coupling time was the same as for standard phosphoramidites. The duration of the oxidation for this step was doubled to ensure the H-phosphonate was fully oxidised before subsequent additions were carried out.

**Cross-link Formation by Copper(I)-Catalysed Azide-Alkyne Cycloaddition**

Reagents were mixed according to the following table, based on a protocol originally obtained from Lumiprobe Ltd. (Russia).

***Table SI-1:*** *Reagent volume (µL) required for various oligonucleotide OD_260_ values for click chemistry.*

|  |  | **Oligo OD_260_** | | | | | | |
| --- | --- | --- | --- | --- | --- | --- | --- | --- |
|  |  | **< 5** | **5 – 10** | **10 – 15** | **15 – 20** | **20 – 25** | **25 – 30** | **30+** |
| **Reagent Volume (µL)** | **Oligo** | 30 | 60 | 90 | 120 | 150 | 180 | + 30 µL |
|  | **2M TEAA Buffer** | 50 | 100 | 150 | 200 | 250 | 300 | + 50 µL |
|  | **10 mM Sodium Ascorbate** | 5 | 10 | 15 | 20 | 25 | 30 | +5 µL |
|  | **CuSO_4_^(a)^** | 5 | 10 | 15 | 20 | 25 | 30 | + 5 µL |
|  | **THPTA^(a)^** | 5 | 10 | 15 | 20 | 25 | 30 | + 5 µL |

1. *THPTA and CuSO_4_ are added as a combined solution containing a 10 mM CuSO_4_/THPTA complex.*

Oligonucleotide was dissolved in 50 µL of Milli-Q water and concentration was measured using UV-Vis spectroscopy (e.g. 7508.1 µM). OD_260_ is the amount of light absorbed by the oligonucleotide when it is suspended in 1 mL of water and measured in a 1 cm cuvette. The value can be calculated by multiplying initial concentration, molar absorptivity, and initial volume of oligonucleotide (e.g., 7508.1 µM × 122800 L mol^-1^ cm^-1^ × 25 µL gives an OD_260_ of 23.05). DNA was then diluted to obtain the reaction concentration: i.e., with an OD_260_ of 23.05 the values in the 20 – 25 column of Table SI-1 were used. The 25 µL of DNA-containing solution is diluted with 125 µL Milli-Q water to give the 150 µL oligonucleotide volume. The corresponding volume of 2 M triethylammonium acetate buffer (pH 7.0, 250 µL) was added to this solution. The 10 mM aq. sodium ascorbate solution was freshly prepared and added, followed by flushing the vial with argon and adding the combined 10 mM aq. CuSO_4_ and 10 mM aq. tris-hydroxypropyltriazolyl-methylamine (THPTA) (50 µl).

Every two hours, 20 µL of reaction mixture was assessed by analytical RP-HPLC for reaction completion until a new peak with shorter retention time was observed. After 4 hours minimal starting material was observed and the reaction mixture was purified by RP-HPLC, collected fractions with absorbance at 260 nm were concentrated by speed-vac and desalted using Nap-5 size exclusion columns.


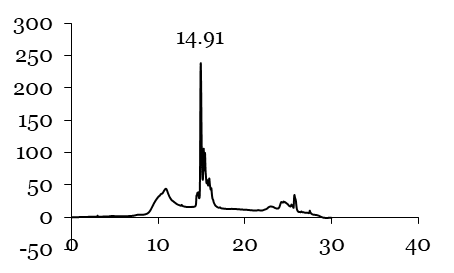

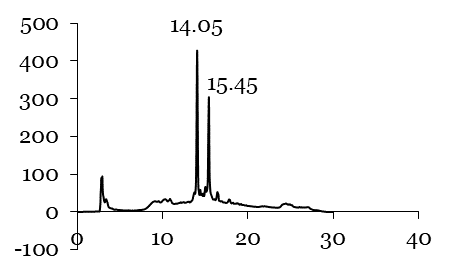

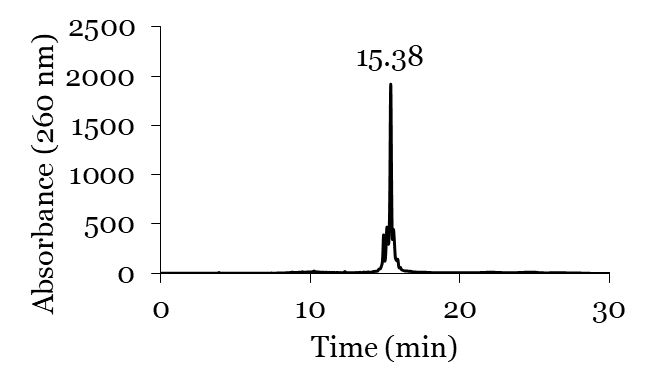

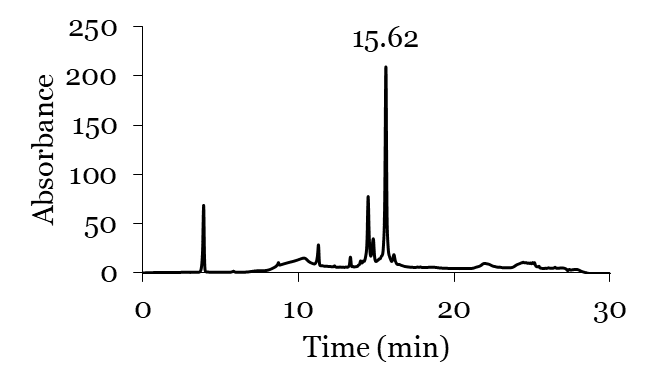


A)

B)

C)

D)


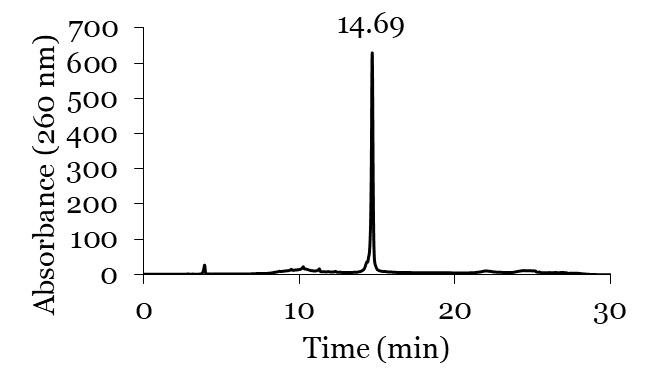

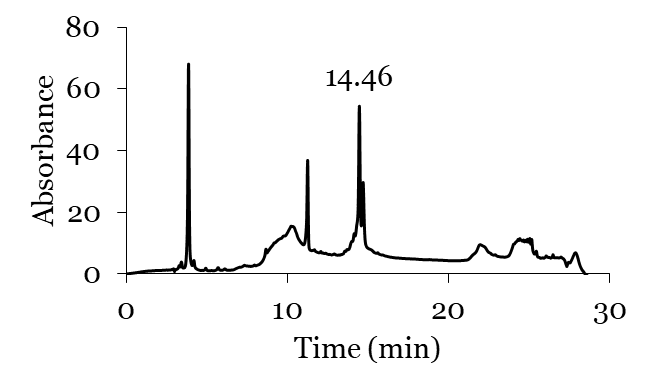


E)

F)

**Figure SI-1**. Comparisons of RP-HPLC profiles of modified tel sequences before, during and after copper-catalysed azide alkyne cycloaddition. A) tel-G3A8 and B) tel-G5A8 before reaction. C) tel-G3A8 and D) tel-G5A8 after 3 hours of cross-linking reaction. E) tel-G5A8-X and F) tel-G5A8-X after purification by RP-HPLC. See Table SI-1 above for reaction conditions. Note: tel-G5A8-X appears to be impure, but the concentration of the oligonucleotide is very low. The peaks observed around 3 and 10 mins are artifacts of the HPLC column used and are also visible in E), although in that case the product peak has much higher intensity.

A)

B)


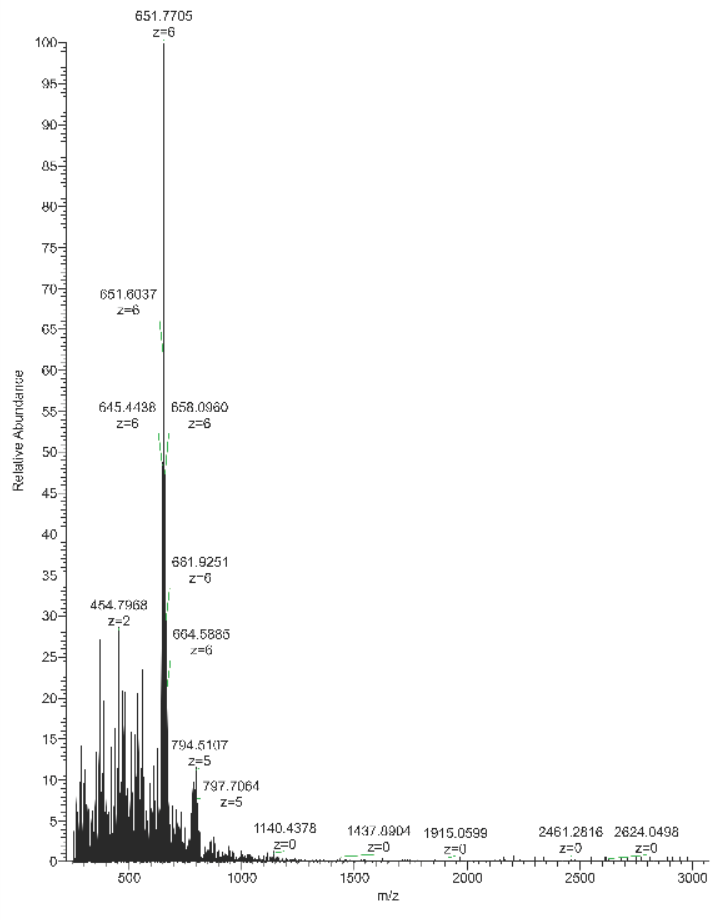

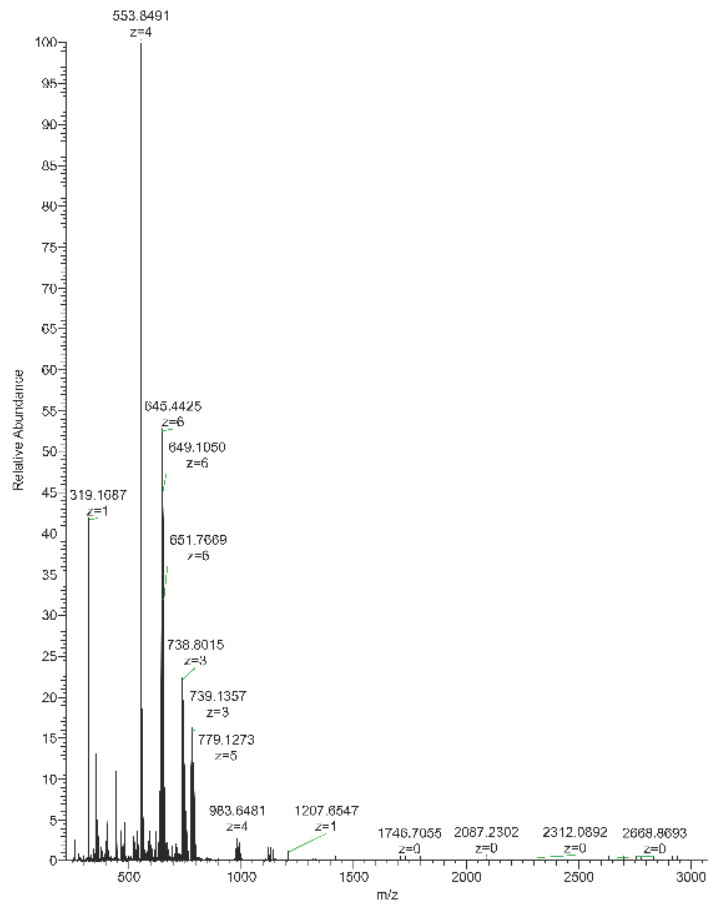


C)

D)

**Figure SI-2.** Comparison of ESI-MS spectra of A) tel-G3A8, B) tel-G3A8-X, C) tel-G5A8 and D) tel-G5A8-X. Before and after cross-linking, the spectra indicate the same mass, meaning the change in retention time observed in RP-HPLC is not due to a difference in sequence.


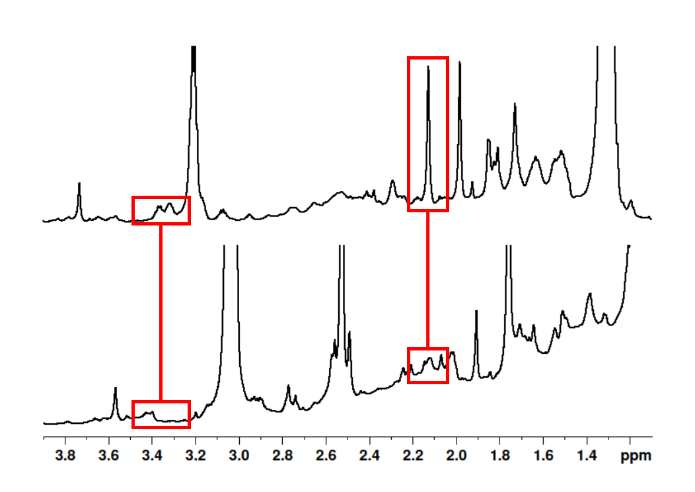


(i)

(ii)

C)

D)


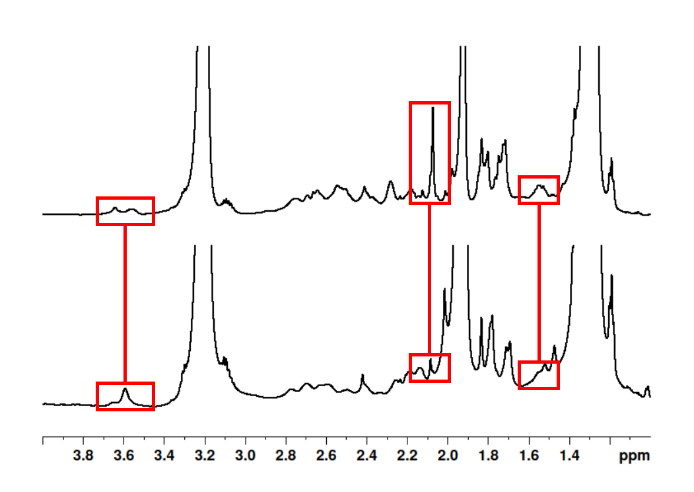


A)

B)

(i)

(ii)

**Figure SI-3**. Comparisons of purified pre- and post-cross-linking modified tel sequences ^1^H NMR. A/B: ^1^H NMR of i) tel-G3A8, ii) tel-G3A8-X A) 1 – 4 ppm and B) 7 – 9 ppm. C/D: ^1^H NMR of i) tel-G5A8, ii) tel-G5A8-X A) 1 – 4 ppm and B) 7 – 9 ppm. Highlighted regions, from left to right, indicate shift in protons corresponding to CH_2_ group adjacent to azide in adenosine modification and disappearance of propargyl proton. Triazole peak could be expected to be visible between 7 and 9 ppm, but this peak is obscured by the dominance of aromatic protons in this region (e.g., H-8 of guanosine or H-8 and H-2 of adenosine). However, significant changes in chemical shift in this region are obvious. Conditions: approx. 200 µM strand concentration, 20 mM sodium phosphate, 10 mM KCl, 10% D_2_O, 1% TSP, pH 7.0, 20 °C.


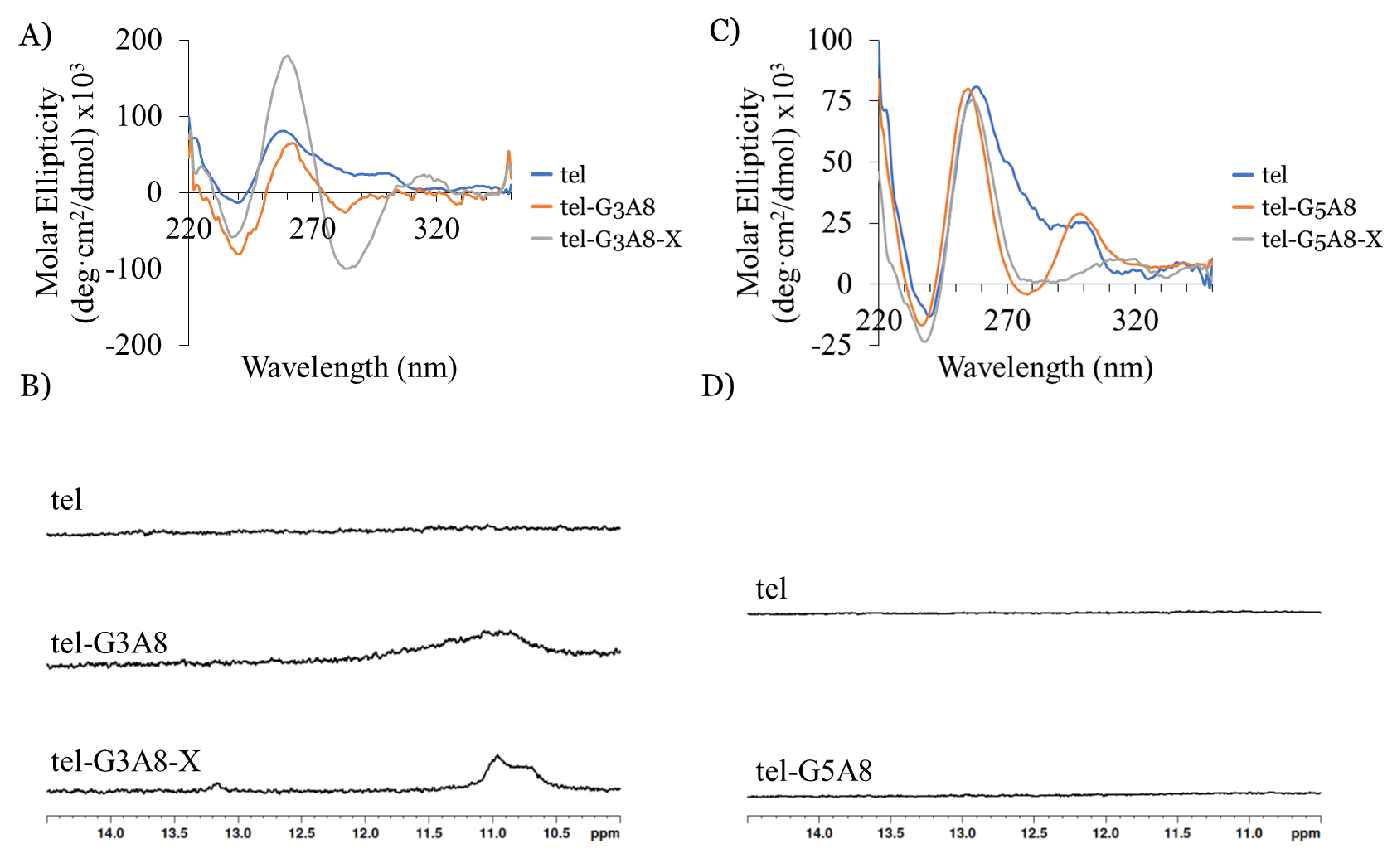


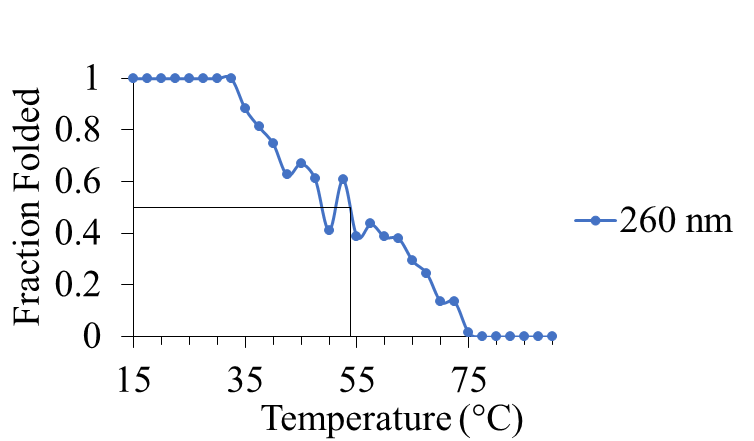

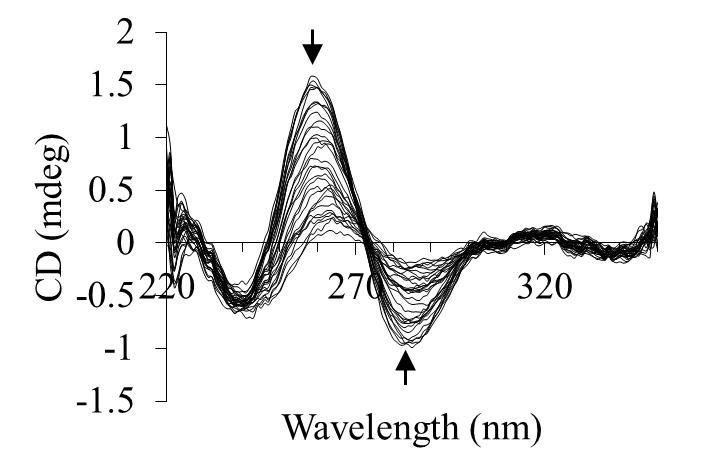

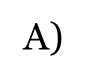

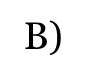


**Figure SI-4.** A) CD spectra and B) imino proton ^1^H NMR spectra of tel, tel-G3A8 and tel-G3A8-X. C) CD spectra and B) imino proton ^1^H NMR spectra of tel, tel-G5A8 and tel-G5A8-X. tel-G5A8-X ^1^H NMR is unchanged from tel-G5A8. Conditions: approx. 20 µM strand concentration (CD) or 200 µM strand concentration (NMR), 20 mM sodium phosphate, 10% D_2_O, 1% TSP, pH 7.0, 20 °C.

**Figure SI-5.** CD melting spectra of tel-G3A8-X in Na^+^ containing buffer. This was the only meltable oligonucleotide in Na^+^ buffer. Conditions: 20 mM sodium phosphate, pH 7.0, heated to 90 °C at 0.625 °C/min. The arrow indicates direction of changes in the peak intensity from low to high temperatures.


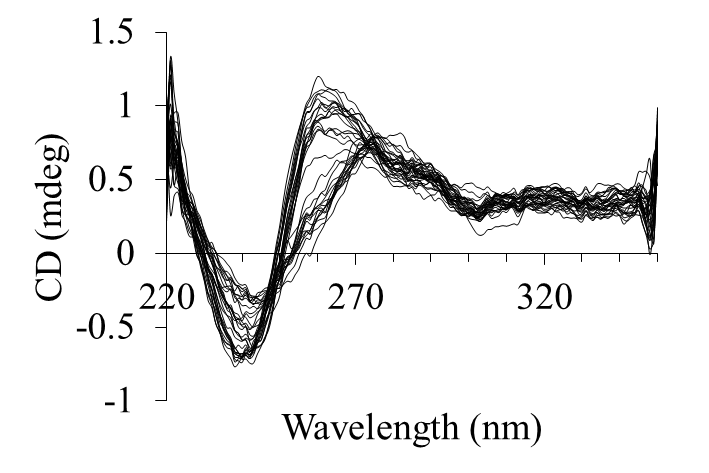

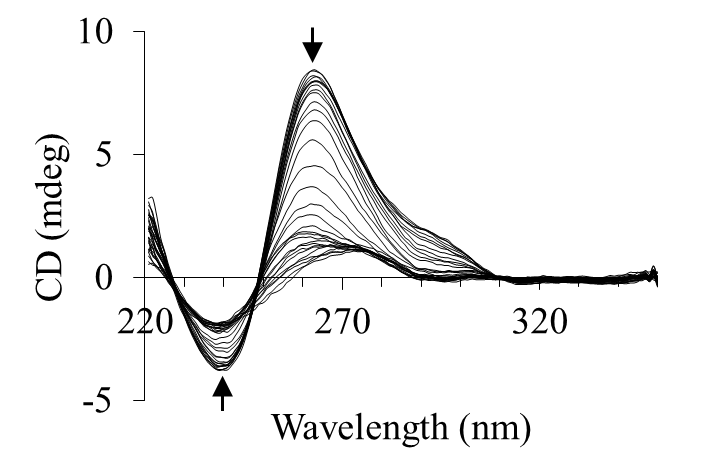

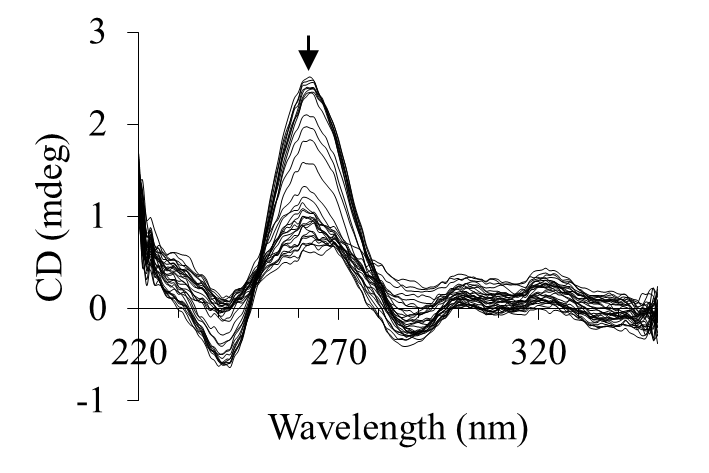

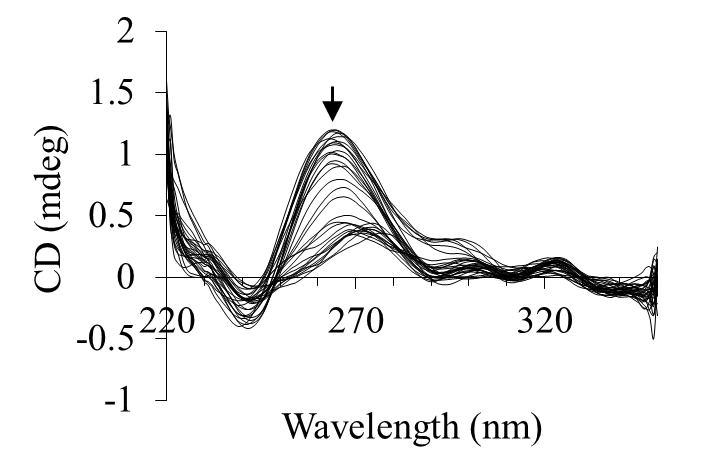

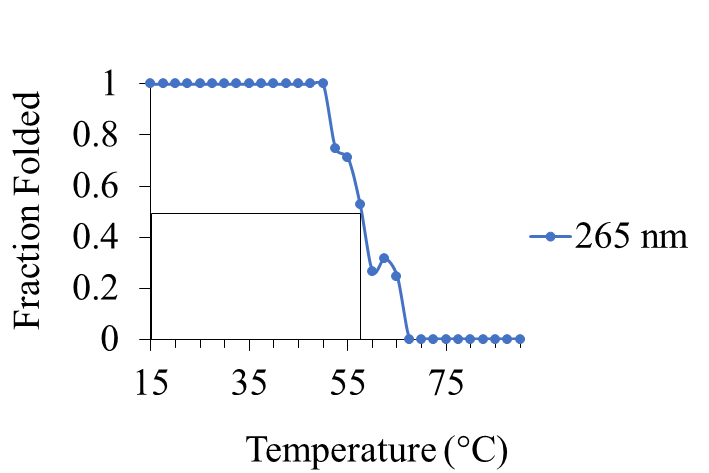

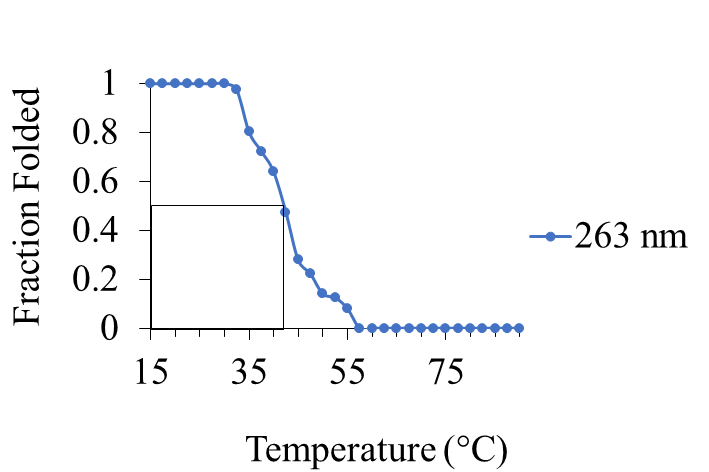

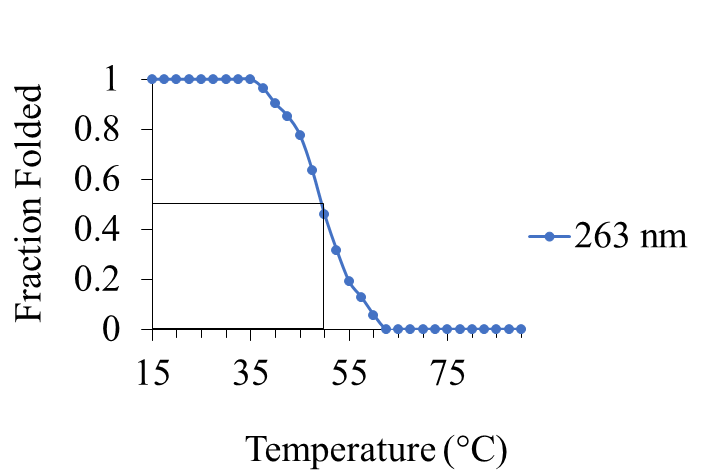


No Melting

A) tel

B) tel-G3A8

C) tel-G3A8-X

D) tel-G5A8


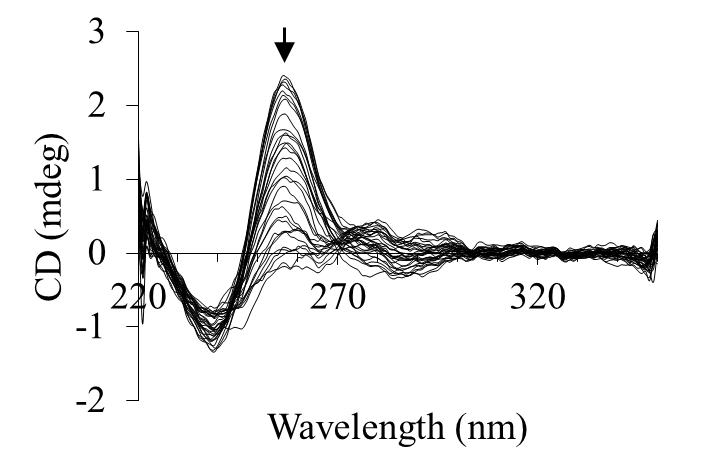


**Figure SI-6.** CD melting spectra of A) tel, B) tel-G3A8, C) tel-G3A8-X, D) tel-G5A8 and E) tel-G5A8-X in K^+^ containing buffer. Conditions: 20 mM sodium phosphate, 10 mM KCl, pH 7.0, heated to 90 °C at 0.625 °C/min. The arrow indicates direction of changes in the peak intensity from low to high temperatures.


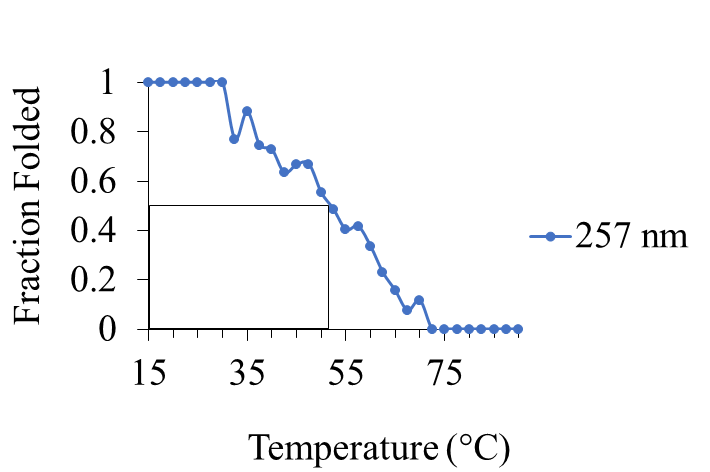


E) tel-G5A8-X


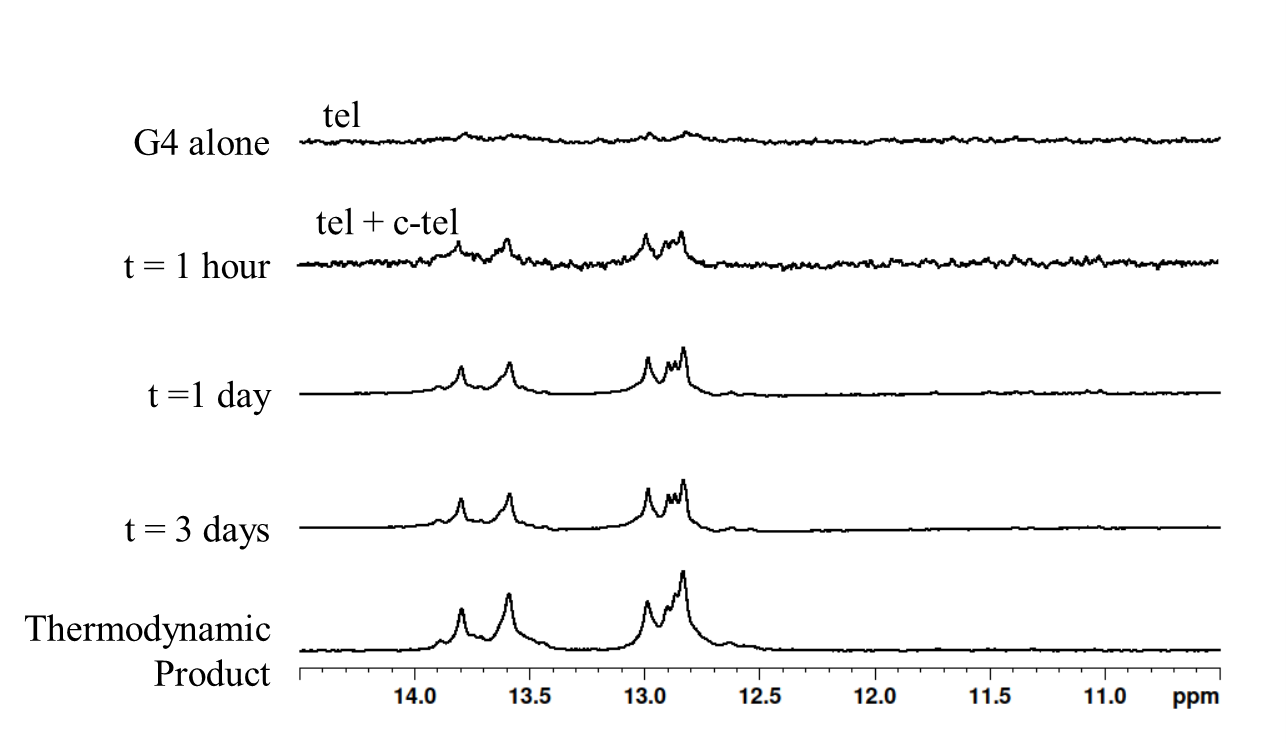


**Figure SI-7**. Imino proton region of ^1^H NMR spectra of unmodified tel sequence challenged with complementary DNA. Conditions: 200 mM strand concentration, 20 mM sodium phosphate, 10 mM KCl, pH 7.0, 20 °C.
